# Combining 3D skeleton data and deep convolutional neural network for balance assessment during walking

**DOI:** 10.3389/fbioe.2023.1191868

**Published:** 2023-06-20

**Authors:** Xiangyuan Ma, Buhui Zeng, Yanghui Xing

**Affiliations:** Department of Biomedical Engineering, Shantou University, Shantou, China

**Keywords:** Kinect, skeleton data, deep convolutional neural network, balance assessment, machine learning

## Abstract

**Introduction:** Balance impairment is an important indicator to a variety of diseases. Early detection of balance impairment enables doctors to provide timely treatments to patients, thus reduce their fall risk and prevent related disease progression. Currently, balance abilities are usually assessed by balance scales, which depend heavily on the subjective judgement of assessors.

**Methods:** To address this issue, we specifically designed a method combining 3D skeleton data and deep convolutional neural network (DCNN) for automated balance abilities assessment during walking. A 3D skeleton dataset with three standardized balance ability levels were collected and used to establish the proposed method. To obtain better performance, different skeleton-node selections and different DCNN hyperparameters setting were compared. Leave-one-subject-out-cross-validation was used in training and validation of the networks.

**Results and Discussion:** Results showed that the proposed deep learning method was able to achieve 93.33% accuracy, 94.44% precision and 94.46% F1 score, which outperformed four other commonly used machine learning methods and CNN-based methods. We also found that data from body trunk and lower limbs are the most important while data from upper limbs may reduce model accuracy. To further validate the performance of the proposed method, we migrated and applied a state-of-the-art posture classification method to the walking balance ability assessment task. Results showed that the proposed DCNN model improved the accuracy of walking balance ability assessment. Layer-wise Relevance Propagation (LRP) was used to interpret the output of the proposed DCNN model. Our results suggest that DCNN classifier is a fast and accurate method for balance assessment during walking.

## 1 Introduction

Balance ability refers to the human body perceiving the body’s center of gravity and controlling the body’s center of gravity within the support plane through body movements (Shumway-Cook et al., 1988). It is crucial for people to maintain their daily activities of living (ADL). Balance impairment may be caused by a number of factors, including advanced age, arthritis, cerebral palsy and Parkinson’s disease, of which advanced age is the most common one. With the growth of age, various functions related to balance abilities decline rapidly, including muscle strength, eyesight, and reaction time, etc. It has been found that as many as 75% of people aged 70 and above have balance disorders (Howe et al., 2011). Balance assessment plays an important role in accurately diagnosing potential disorders, identifying fall risks, and developing treatment plans. Currently, balance assessment is usually done by physical therapists based on commonly used standardized measures, such as Berg balance scale, Tinetti balance scale and timed “up & go” test (TUG).

Walking balance assessment is an important part in a variety of balance standardized measures. For example, in Berg balance scale, subjects are required to walk from one position to another, and the physical therapist will give them scores based on their walking stability, gait and posture. In Tinetti balance scale, foot and trunk positions as well as walking path and time of subjects are considered. Similarly, the TUG test mainly use walking speed as an index to judge person’s balance abilities and fall risks.

Balance ability scores in balance standardized measures are assessed based on judgments from the physical therapists, which is subjective. To address this issue, one way is to analyze the balance abilities with equipment-based and digitalized assessment methods in order to eliminate subjective opinions from physical therapists. In equipment-based and digitalized assessment methods, the motion of human body can be described with 3D skeleton data. Thus, it is possible to use 3D skeleton data to assess balance abilities during walking.

3D skeleton data can be acquired in several ways, such as Microsoft Kinect, Orbbec Astra, and Intel RealSense. Microsoft Kinect is the most popular method. Kinect can recognize and track a total of 32 human joints, covering all parts of the human body. Currently, there are a number of relevant studies on the accuracy of Kinect. Eltoukhy (Eltoukhy et al., 2017) simultaneously used Kinect and Vicon system to record the results of Star Excursion Balance Test (SEBT), and found that the kinematic error of lower limbs was less than 5° except for the front plane angle of the posterior and lateral knee joints, which were 5–7°. According to Schmitz (Schmitz et al., 2014), the accuracy and precision of joint angle measurement in 3D skeleton model obtained from Kinect are equivalent to that of the mark-based system. Khoshelham (Khoshelham, 2012) believes that the point cloud data of Kinect sensor is able to provide acceptable accuracy by comparing it with the point cloud data of high-end laser scanner. Kinect has the characteristics of low cost, portability and convenient data access, and is able to provide acceptable accuracy.

At present, there are plenty of studies on gait recognition, pathological gait classification and motion analysis using Kinect 3D skeleton data (Ahmed et al., 2014; Li et al., 2018; Bari and Gavrilova, 2019; Jun et al., 2020; Xing and Zhu, 2021), all of which have acceptable accuracy. However, further process is needed in order to assess balance abilities during walking. Balance abilities assessment during walking not only need to observe the gait, but also need to consider walking speed and other parameters. The existing methods so far are mainly designed for gait recognition and motion analysis. In this study, we aim to develop a digitalized assessment method for assessing balance abilities during walking based on 3D skeleton data collected by Kinect.

Deep convolutional neural network (DCNN) is a popular machine learning technology, and has excellent performance in the fields of image recognition (Szegedy et al., 2015; Senior et al., 2020; Li et al., 2021). Also, DCNN classifier is widely used in data and image analysis applications (Yao et al., 2019; Yu et al., 2019; Szczęsna et al., 2020; Yu et al., 2022a; Yu et al., 2022b). DCNN can achieve automatic feature extraction based on original sensor data (Alharthi et al., 2019). One of the limitations of DCNN is its opacity (Bach et al., 2015). This opacity seriously hinders the acceptance and application of DCNN model in medical diagnosis (Wolf et al., 2006). To solve this problem, Layer-wise Relevance Propagation (LRP) was proposed (Montavon et al., 2017), which uses the back propagation method to attribute part of the model prediction to the original input signal. It is possible to identify the important area of classification hoisting in the input signal, which may be used to interpret the prediction of the model. Currently, LRP has been successfully applied in image classification (Lapuschkin et al., 2017), text classification (Arras et al., 2017) and gait analysis (Horst et al., 2019).

In this study, we proposed a deep learning-based method for balance assessment during walking based on 3D skeleton data collected by Kinect. The main contributions and innovation of this study are listed as follows.

(1) Existing Kinect-based methods are only designed for gait recognition and motion analysis. We newly collected a Kinect 3D skeleton dataset with three standardized balance ability levels and specifically designed a deep learning based method for balance ability assessment during walking.
(2) To obtain better performance, we performed thorough parametric study including hyperparameters setting of DCNN and skeleton node selection. We also used LRP to interpret the output of the DCNN model.
(3) To validate the performance of the proposed method, we conducted a comparison with other commonly used machine learning methods. In addition, we migrated and applied a state of art posture classification method to the walking balance ability assessment task. The proposed method was also compared with the migrated state of art method. The results showed the superiority of the proposed method.

## 2 Materials and methods

### 2.1 Experimental subjects and balance impairment simulation

**FIGURE 1:**
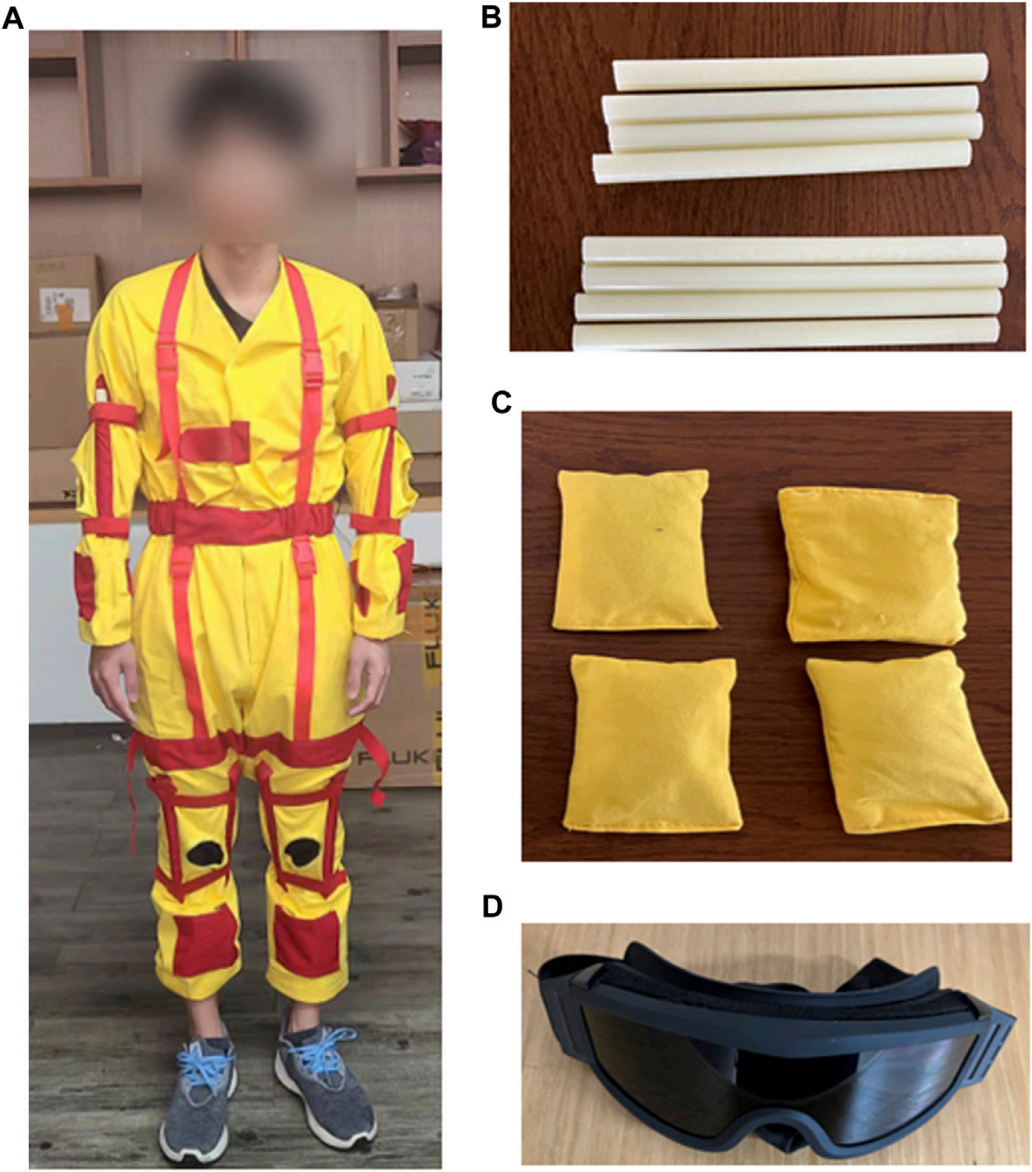
Age simulation suit. **(A)**: Subject with age simulation suit; **(B)**: sticks used to restrict knee and elbow joints; **(C)**: Sandbags located on the limbs; **(D)**: Special glasses to simulate vision loss.

We recruited ten healthy adults aged from 21–24 as our experimental subjects, who have normal balance abilities. For balance impaired subjects, we asked the ten adults wearing age simulation suits, which are widely used for healthy people to experience functional challenges of the elderly. After wearing the suits, the young adults will have reduced motor and cognitive performance, as well as self-perception ability (Saiva et al., 2020; Vieweg and Schaefer, 2020). Additionally, the suit users will also experience a variety of other functional disabilities including reduced muscle strength, vision loss, decreased flexibility of the joints (Lauenroth et al., 2017; Bowden et al., 2020; Watkins et al., 2021), which can lead to the decline in balance as in older people. The simulation suit is shown in Figure 1. Briefly, we use wrist and ankle weights to simulate loss of muscle strength in the extremities, and use knee and elbow adjustments to limit joint flexibility. We also can adjust two ropes in front of the chest to simulate hunchback status, and tie the rope around thighs to limit step width. Additionally, special glasses were used for poor vision simulation.

There are two setups of the suit to simulate two different types of balance disabilities that may happen during the aging process. Thus, for one healthy subject, we can acquire totally three standardized balance levels. Under the guidance of rehabilitation doctors, our simulation guidelines are as follows:

- Level-0: No restrictions are added, subjects walk normally.
- Level-1: Subjects are given extra weight on their wrists and ankles, and have limited knee and elbow flexibility.
- Level-2: On the basis of level-1, the conditions of hunchback and limited vision are added.

### 2.2 Data collection

**FIGURE 2:**
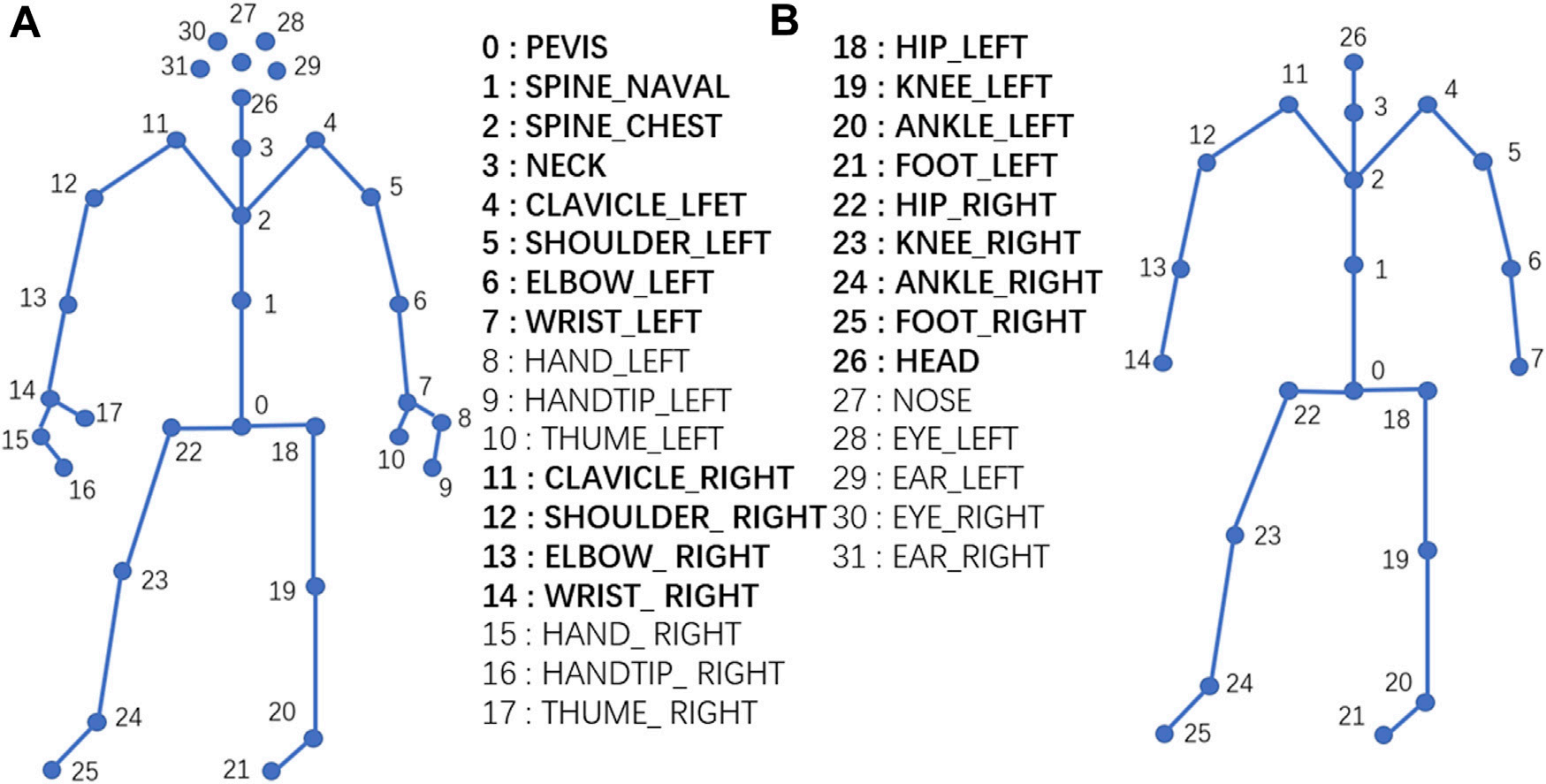
Joint diagram of human skeleton. **(A)**: The original skeleton structure from Kinect with 32 joints; **(B)**: Simplified skeleton structure for this study with 21 joints in bold font.

Following simulation guidelines, every subject walked 10 times for each of the balance levels. Thus, the size of the dataset is 300 (10 subjects × 10 times × 3 balance levels). 3D skeleton data were obtained using a Kinect sensor and the corresponding software development kit named Kinect SDK. The skeleton data includes 3D coordinates of total 32 joints, and the joint hierarchy is distributed in a direction from the center of the body to the extremities. Joints includes pelvis, shoulder, elbow, wrist, hand, hang tip, thumb, hip, knee, ankle, foot, head, nose, eye, and ear, as shown in Figure 2A. Based on our preliminary studies, some of these joints including hand, hang tip, nose, eye and ear have minor impact on balance abilities, and were removed in this research. The final skeleton structure for data collection is shown in Figure 2B.

**FIGURE 3:**
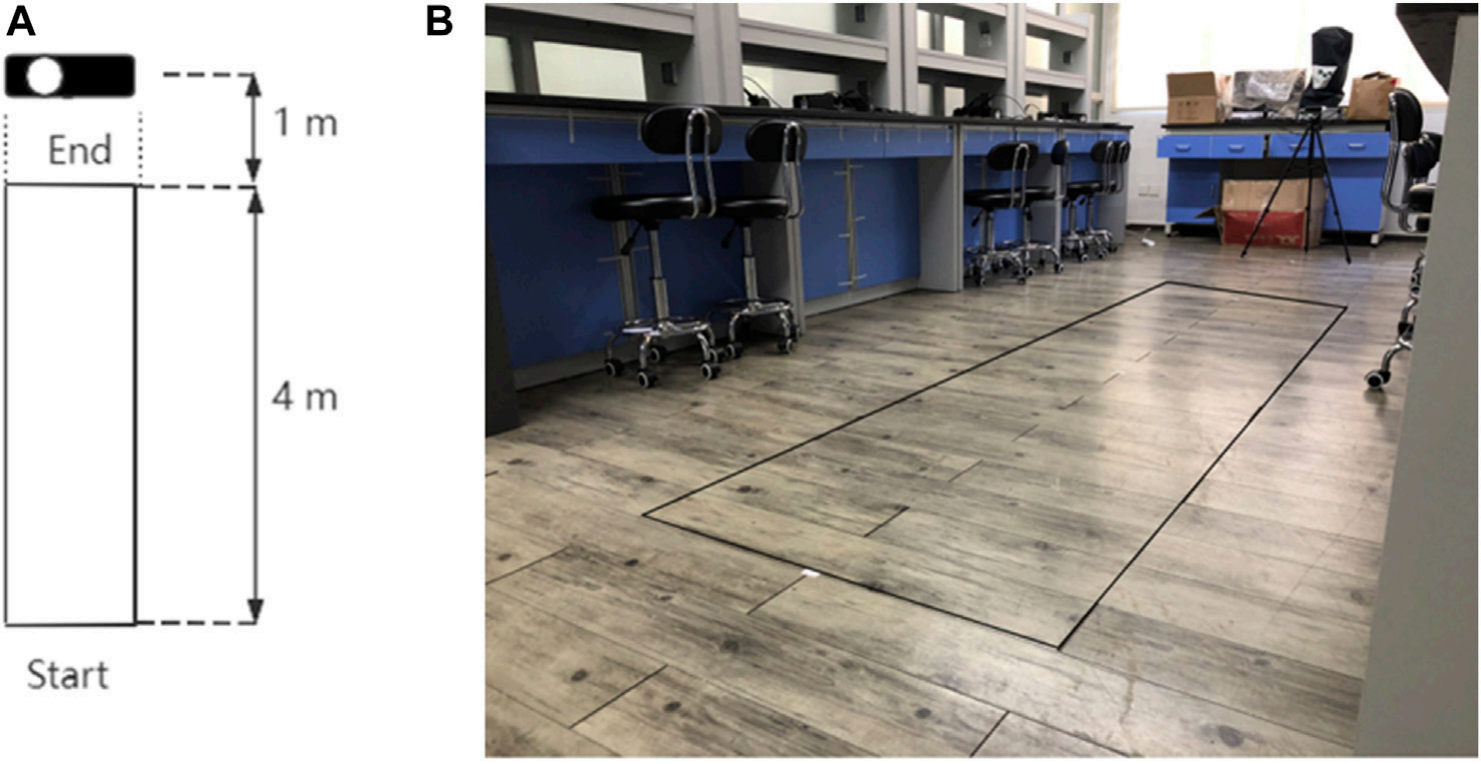
Data acquisition process with Kinect. **(A)**: Abstract figure of scene; **(B)**: Real scenarios of data acquisition process.

The data acquisition process by Kinect is shown in Figure 3. We set up a 4-meter long sidewalk, and the two ends of the sidewalk are the starting point and the end point respectively. The Kinect device is located 1 meter away from the end point. Subjects are required to walk from the starting point to the end point at their normal speed.

### 2.3 Deep convolutional neural network (DCNN)

Deep convolutional neural networks are frequently used for classification and recognition tasks and show great performance; therefore, some researchers also use DCNN to process 3D skeleton data (Caetano et al., 2019; Li et al., 2020). RNN, LSTM and GRU have been proposed successively for processing sequence data [text (Cho et al., 2014), audio (Yue-Hei Ng et al., 2015), video (Ballas et al., 2015)]. Because 3D skeleton data is time series sequence data, these three models can be used to process 3D skeleton data (Graves et al., 2013; Lee et al., 2019). In addition, as the human skeleton connected by the junction nodes is similar to the graph in the computer data type, there are some studies on 3D skeleton data analysis based on graph convolution network (Cheng et al., 2020; Shi et al., 2020). In summary, neural network models commonly used for skeleton data processing include DCNN, RNN and GCN. In this paper, we use convolutional networks to construct a model for processing the hierarchical assessment of dynamic balancing capability based on 3D skeleton data.

The core building module of deep convolutional network model is the convolution layer, which mainly extracts features by applying convolution operation. Assuming that X is the input of the convolution layer, W is the weight matrix, and B is bias, the output Y of the convolution layer can be expressed as:

$$Y=Conv\left(W,X\right)+b$$

An activation function φ is usually added after the convolution operation:

$$Y=\varphi \left(Y\right)$$

**FIGURE 4:**
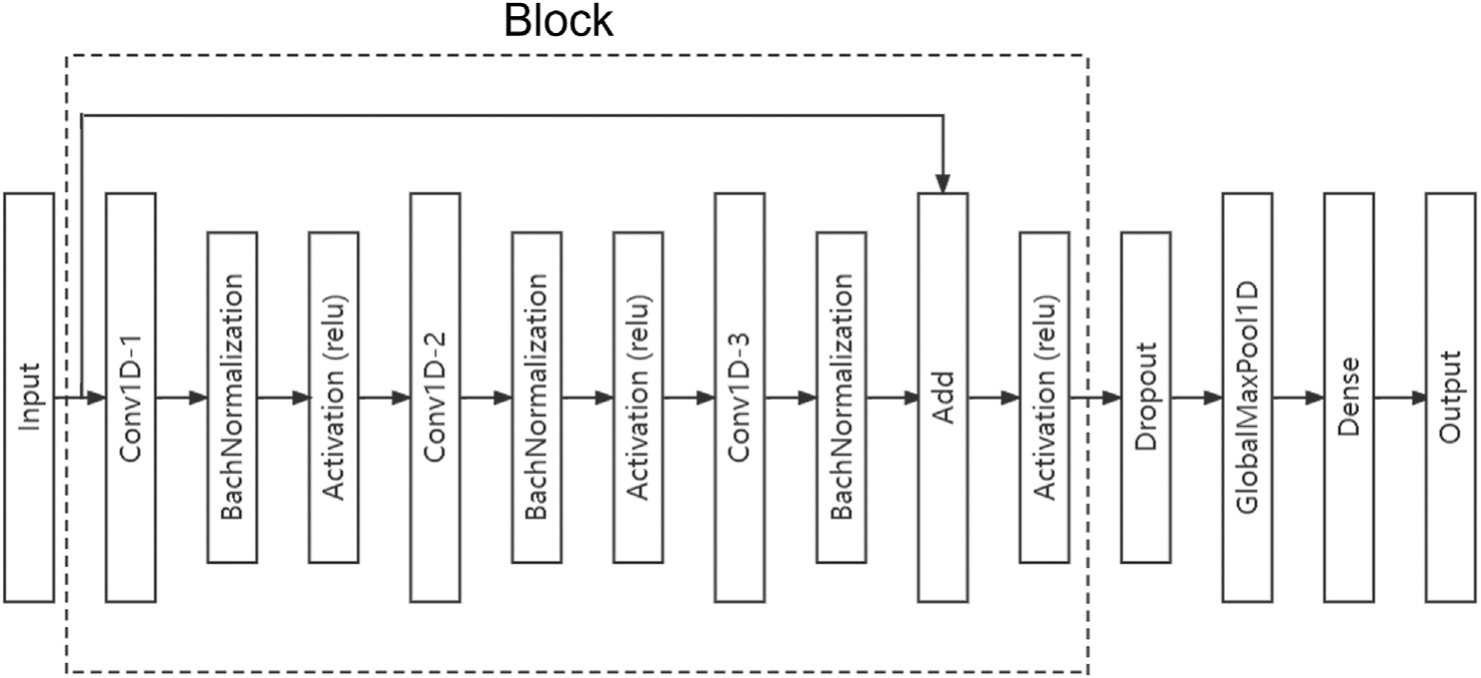
Proposed DCNN structure. DCNN mainly consists of a single or multiple residual blocks, each of which includes three convolutional layers, batch normalization layers, and Relu activation layers. We can add multiple residual blocks between input layer and dropout layer if needed.

The structure of the DCNN constructed in this paper is shown in Figure 4. The model consists of several residual blocks, a dropout layer, a global pooling layer and a full connection layer. Residual block is mainly composed of three convolution layers and a connection layer. Convolution layer 1 processes the input data, convolution layer 2 processes the output of convolution layer 1, convolution layer 3 processes the output of convolution layer 2, and then the connection layer merges the input of convolution layer 1 and the output of convolution layer 3. Batch Normalization and activation using Relu are required following each convolutional layer in a convolution block. In a residual block, the size of convolution kernel of convolution layer 1 is set as 5, the size of convolution kernel of convolution layer 2 is set as 3, and the size of convolution kernel of convolution layer 3 is set as 1.

### 2.4 Layer-wise relevance propagation (LRP)

Linear models make transparent decisions. However, complex nonlinear models are usually regarded as black box classifiers, and almost all deep artificial neural networks are composed of nonlinear models. Layer-wise Relevance propagation (LRP) is a technique for generalized interpretation of nonlinear models. We used LRP to interpret the output of the proposed DCNN model in this paper.

The LRP (Montavon et al., 2017) uses the topology of the network model itself to attribute the correlation score to the important components of the input data, so as to explain the decisions made by the model based on a given data point. Based on the conservation principle, LRP technology (Bach et al., 2015) gradually maps the prediction to a lower layer through back propagation until the input variable is reached. Neuron j receives a quantity of relevance $R_{j}^{l+1}$ from upper layer neurons, and redistributes that quantity to neuron i , in proportion to $R_{i\leftarrow j}^{\left(l,l+1\right)}$ (the contribution of neuron i to the activation of neuron j in the forward pass).

$$R_{i\leftarrow j}^{\left(l,l+1\right)}=\frac{z_{ij}}{z_{j}}∙R_{j}^{l+1}$$

Where $z_{ij}$ represents the measurement of the contribution from neuron i to neuron j , and $z_{j}$ represents the sum of all neurons from l layer to neuron j .

### 2.5 Model training

#### 2.5.1 Pre-processing of input samples

The time length of each video sample recorded in the dataset was inconsistent because different subjects may have different walking speeds. Thus, in the temporal aspect, the total number of frames of different samples was different. The total number of frames of different samples collected in this study varied from 22 frames to 38 frames.

To normalize the DCNN input size, we selected fixed 20 frames for every sample in the dataset from back to front. Occasionally, subjects did not walk immediately after receiving the command, selected from back to front could avoid the collection of few non-walking frames. Besides, since the sample data may be offset, we reoriented the input data of each sample based on the position of the pelvis joint in the first frame of each sample. Specifically, for each data point in each sample, its position coordinates need to subtract the pelvis joint in the original coordinates.

#### 2.5.2 Training-validation-test split

**FIGURE 5:**
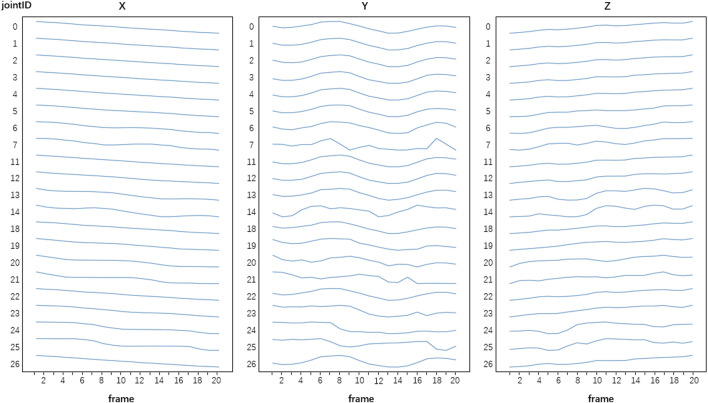
An example of a pre-processed sample. Each sample has data of 21 joints, 20 frames and three-dimensional (X, Y, Z) time-varying spatial coordinates of the joints after pre-processing.

In this study, we obtained a dataset of 300 samples. After data pre-processing, the total number of frames of each sample was 20 frames, each frame had data of 21 human joints, and each joint had data of three-dimensional (X, Y, Z) time-varying spatial coordinates. Thus the size of an input sample was 1,260 (20 frames × 21 joints × 3 dimensions). Figure 5 show an example of a pre-processed input sample.

Since it was a relatively small dataset, we adopted leave-one-subject-out-cross-validation strategy to train and evaluate the performance of the DCNN. In each fold, one subject data was used as validation dataset, another one subject data was used as test dataset and the remaining eight subject data was used as training dataset. Finally, the size of training dataset was 240 (8 subjects × 10 times × 3 balance levels), the size of validation dataset was 30 (1 subjects × 10 times × 3 balance levels), and the size of test dataset was 30 (1 subjects × 10 times × 3 balance levels) in each fold.

#### 2.5.3 DCNN hyperparameters setup

After parametric study, for the residual block, we set the size of kernel of residual block in the first convolutional layer to 5, the size of kernel in the second convolutional layer to 3, the size of kernel in the third convolutional layer to 1. The padding method was same, and the activation function was Relu function in every convolutional layer. The channel size of filter in convolution layer is an important parameter, we chose 64 after comparing with other size. For epoch selection, we applied the early stop method. The early stop method is a widely used method to stop training when the performance of the model on the validation dataset begins to decline, so as to avoid the problem of overfitting caused by continued training. In addition, cross-entropy was chose as the loss function and Adam optimizer with a learning rate of 0.0001 was used to train DCNN classifier.

#### 2.5.5 Performance evaluation measures

Accuracy, Precision and F1-Score were used to measure the model, and the results from 10 test folds were then pooled together to form a complete set to calculate the average values of the measures. Accuracy, Precision and F1-score are counted by the true positive (TP), false positives (FP), true negatives (TN), and false negatives (FN). In multi-classification tasks, Precision and F1 indices need to be calculated for each category, and then arithmetic average is performed.

$$Accutacy=\frac{TP+TN}{TP+FP+TN+FN}$$

$$Precision=\frac{TP}{TP+FP}$$

$$F_{1}=2∙\frac{Precision∙Recall}{Precision+Recall}$$

Since artificial neural networks are often seen as black boxes, methods such as SmoothGrad (Smilkov et al., 2017), Deconvnet (Springenberg et al., 2015), GuidedBackprop (Alber et al., 2018), and LRP (Montavon et al., 2017) have been proposed to remedy this deficiency. INNvesigate is a library that implements the PattNet, PatternAttribution (Kindermans et al., 2017), and LRP methods, allowing the user to invoke them through the interface. We called LRP method based on iNNvesigate for 3D skeleton data analysis.

## 3 Results and discussion

### 3.1 Performance of proposed DCNN for balance assessment during walking

We use the proposed DCNN method to classify three different levels of balance ability during walking. With leave-one-subject-out cross-validation, the method achieved 93.33% accuracy, 94.44% precision and 94.46% F1 score. The results showed that Kinect can collect 3D skeleton data to assess the balance ability of walking subjects, and the accuracy of DCNN classification can reach an accepted level.

**FIGURE 6:**
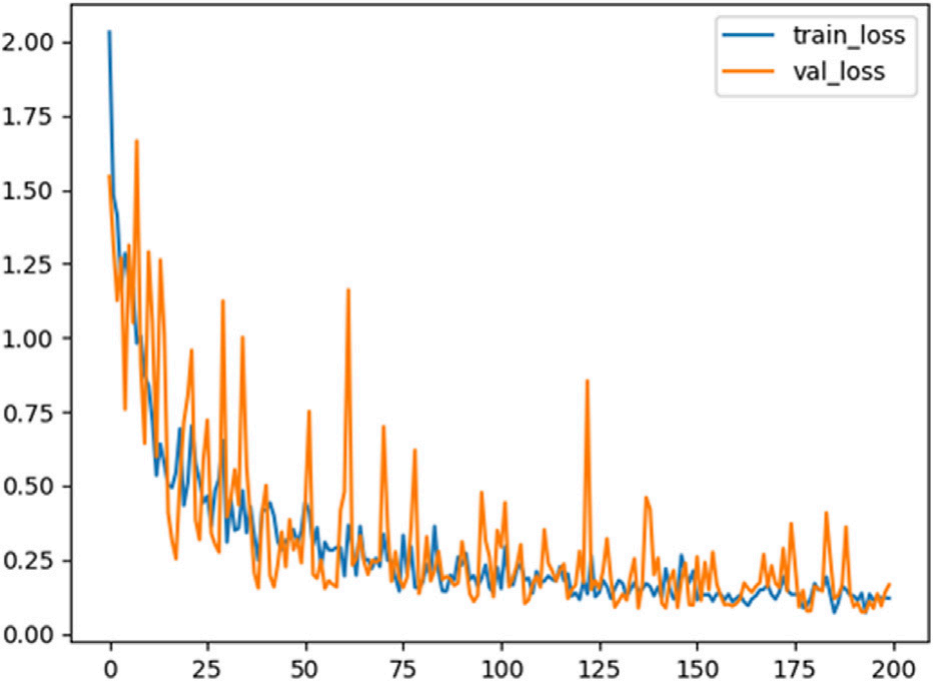
Loss function variation curve of training set and validation set. The blue line represents the loss function curve of the training dataset and the orange line represents the loss function curve of the validation dataset.

The loss function of balance ability classification based on the DCNN model is shown in Figure 6, where the blue line represents the loss function curve of the training dataset and the orange line represents the loss function curve of the validation dataset. We found that when Adam optimizer with learning rate of 0.0001 was used for training, the loss function value of DCNN classifier decreased as the number of iterations increased, and stabilized after 150 epochs, with a fluctuation of 0.25. The result suggests that DCNN classifier is convergent.

### 3.2 Parametric study of DCNN hyperparameters selection

#### 3.2.1 Kernel size

Kernel size is an import parameter in convolution layer. The larger the convolution kernel is, the larger the receptive field is. However, a large convolution kernel will lead to a huge increase in the amount of computation and may reduce the computational performance. We compared the performance of DCNN with different kernel size and the results were showed in Table 1. After comparing the performance of DCNN with different kernel size in the residual block, we set the size of kernel in the first convolutional layer to 5, the size of kernel in the second convolutional layer to 3, the size of kernel in the third convolutional layer to 1.

**TABLE 1:** The validation loss and the validation accuracy with different kernel size.

Kernel size 1	Kernel size 2	Kernel size 3	Validation loss	Validation accuracy
1	1	1	0.2424	0.9426
1	1	3	0.2460	0.9444
1	1	5	0.2128	0.9519
1	1	7	0.2316	0.9444
1	3	1	0.2098	0.9556
1	3	3	0.2060	0.9630
1	3	5	0.1982	0.9593
1	3	7	0.2093	0.9556
1	5	1	0.1840	0.9630
1	5	3	0.1720	0.9704
1	5	5	0.2038	0.9519
1	5	7	0.1995	0.9481
1	7	1	0.2243	0.9444
1	7	3	0.1823	0.9630
1	7	5	0.1696	0.9667
1	7	7	0.1493	0.9626
3	1	1	0.2045	0.9630
3	1	3	0.2050	0.9556
3	1	5	0.1436	0.9778
3	1	7	0.2299	0.9704
3	3	1	0.1974	0.9556
3	3	3	0.1649	0.9704
3	3	5	0.2303	0.9481
3	3	7	0.1893	0.9667
3	5	1	0.1811	0.9556
3	5	3	0.1701	0.9667
3	5	5	0.1556	0.9704
3	5	7	0.2170	0.9556
3	7	1	0.1917	0.9667
3	7	3	0.1465	0.9715
3	7	5	0.2123	0.9593
3	7	7	0.1636	0.9667
5	1	1	0.1677	0.9704
5	1	3	0.2052	0.9630
5	1	5	0.1848	0.9630
5	1	7	0.1757	0.9593
5	3	1	0.1420	0.9778
5	3	3	0.2075	0.9667
5	3	5	0.1930	0.9667
5	3	7	0.1795	0.9667
5	5	1	0.1983	0.9667
5	5	3	0.2169	0.9630
5	5	5	0.2137	0.9630
5	5	7	0.2030	0.9593
5	7	1	0.1930	0.9667
5	7	3	0.2082	0.9444
5	7	5	0.1874	0.9630
5	7	7	0.1538	0.9635
7	1	1	0.1956	0.9667
7	1	3	0.1654	0.9630
7	1	5	0.1986	0.9593
7	1	7	0.1823	0.9704
7	3	1	0.2001	0.9593
7	3	3	0.1816	0.9741
7	3	5	0.1599	0.9704
7	3	7	0.1658	0.9630
7	5	1	0.1669	0.9667
7	5	3	0.1489	0.9704
7	5	5	0.1485	0.9630
7	5	7	0.1649	0.9715
7	7	1	0.2015	0.9630
7	7	3	0.1393	0.9615
7	7	5	0.1988	0.9667
7	7	7	0.1816	0.9593

#### 3.2.2 Channel size

Channel can usually be understood as the width of model, and increasing the width allows each layer to learn richer features. However, increasing the number of channels may also affect the performance of the model. Table 2 shows the impact of different channel size on model performance. We can see that the loss function of validation dataset shows a downward trend, and the accuracy of validation dataset shows an upward trend.

**TABLE 2:** The validation loss and the validation accuracy with channel size.

Channel size	Validation loss	Validation accuracy
4	0.2455	0.9555
8	0.2196	0.9518
16	0.2215	0.9518
32	0.1489	0.9704
64	0.1411	0.9852

#### 3.2.3 The number of residual blocks

The number of residual blocks is also an important parameter for DCNN. Usually, the more residual blocks, the deeper the DCNN model will be, and the more accurate classification model can be fitted. However, as the depth of the model increases, it will be easy to learn the noise of input data, resulting in the phenomenon of overfitting. Table 3 shows the impact of different number of residual blocks on model performance. We can see that, as the number of residual blocks increases, the training loss value decreases, but the validation loss decreases first and then increases. When the number of residual blocks is 3, the validation loss of DCNN model reaches the minimum value, which is 0.1539, and the loss accuracy is 0.9741.

**TABLE 3:** Performance of DCNN with different number of residual blocks.

The number of residual blocks	Train loss		Validation loss	Validation accuracy
1	0.1386		0.1663	0.8333
2	0.1237		0.1569	0.9556
3	0.1235		0.1539	0.9741
4	0.1001		0.1856	0.9667

#### 3.2.4 Optimizer

In order to obtain better performance, we use different optimizers for optimization analysis. Stochastic Gradient Descent (SGD) algorithm randomly selects a group of samples from each iteration and updates them according to the Gradient after training. Root Mean Square Propagation (RMSProp) is an exponential movement-weighted average of the binary norm of each component of the historical gradient. Adam Optimizer takes advantage of the advantages of AdaGrad and RMSProp optimizer and is considered to be quite robust to the selection of hyperparameters. Table 4 shows the performance of DCNN with different optimization methods. From Table 4, we can see that although the average epoch of Adam optimizer is larger than the others, its validation loss is smaller than the others, and fitting effect is better.

**TABLE 4:** Performance of DCNN with different optimization methods.

Optimizer	Epoch	Validation loss	Validation accuracy
SGD	79	0.3040	0.9184
RMsProp	80	0.2982	0.9259
Adam	95	0.1539	0.9741

### 3.3 Parametric study of skeleton node selection

In order to improve the performance of the proposed DCNN classifier, we also tried to optimize skeleton node selection and subsequently to control the data input to the model. We have previously excluded some nodes (fingers, ears, and nose) in the process of data collection, but not all the remaining nodes have positive effects on the assessment of balance ability during walking. The body’s joints are connected with each other, and some joints are dependent on the existence of other joints. For example, if we exclude elbow from input data, we should also exclude wrist data. Following this principle, we observed the performance change of DCNN classifier by selectively inputting data of specific nodes, the results were shown in Table 5.

**TABLE 5:** Performance of different joint groups.

Group	Description	Selected joints	Accuracy	Improvement
A0	All joints	0, 1, 2, 3, 4, 5, 6, 7, 11, 12, 13, 14, 18, 19, 20, 21, 22, 23, 24, 25, 26	89.83	
T1	Joints except for head	0, 1, 2, 3, 4, 5, 6, 7, 11, 12, 13, 14, 18, 19, 20, 21, 22, 23, 24, 25	86.34	−3.49
T2	Joints expect for head, neck	0, 1, 2, 4, 5, 6, 7, 11, 12, 13, 14, 18, 19, 20, 21, 22, 23, 24, 25	86.67	−3.16
H1	Joints except for wrists	0, 1, 2, 3, 4, 5, 6, 11, 12, 13, 18, 19, 20, 21, 22, 23, 24, 25, 26	88.67	−1.16
H2	Joints except for wrists, elbows	0, 1, 2, 3, 4, 5, 11, 12, 18, 19, 20, 21, 22, 23, 24, 25, 26	91.33	1.50
H3	Joints except for wrists, elbows, shoulders	0, 1, 2, 3, 4, 11, 18, 19, 20, 21, 22, 23, 24, 25, 26	91.00	1.17
H4	Joints except for wrists, elbows, shoulders, clavicles	0, 1, 2, 3, 18, 19, 20, 21, 22, 23, 24, 25, 26	93.67	3.84
L1	Joints expert for feet	0, 1, 2, 3, 4, 5, 6, 7, 11, 12, 13, 14, 18, 19, 20, 22, 23, 24, 26	89.00	−0.83
L2	Joints expert for feet, ankles	0, 1, 2, 3, 4, 5, 6, 7, 11, 12, 13, 14, 18, 19, 22, 23, 26	88.67	−1.16
L3	Joints expert for feet, ankles, knees	0, 1, 2, 3, 4, 5, 6, 7, 11, 12, 13, 14, 18, 22, 26	87.33	−2.50
L4	Joints expert for feet, ankles, knees, hips	0, 1, 2, 3, 4, 5, 6, 7, 11, 12, 13, 14, 26	84.33	−5.50
G1	Joints only include pelvis, spine naval, spine chest	0, 1, 2	76.00	−13.83
G2	Joints only include feet, ankles, knees, hips	18, 19, 20, 21, 22, 23, 24, 25	67.33	−22.50

We take the inputs of all joints as the control group (A0), and the classification accuracy was 89.83%. We found that the accuracy of the classifier increased when we excluded the hand-related data. Specifically, when the wrist and elbow (H2) were excluded, the accuracy of the classifier increased to 91%. When wrists, elbows, shoulders and clavicles were excluded (H4), the accuracy of the classifier increased to 93.67%. However, the accuracy of the classifier decreased after excluding leg-related joints. When foots, ankle, knees and hips were excluded (L4), the classifier’s accuracy fell to 84.33%. Furthermore, when using only the pelvis, spine naval, spine chest (G1) data, the classifier has only 76% accuracy. When using only leg-related data, including feet, wart-form, knees and hips (G2), the classifier achieved 67.33% accuracy. The results suggest that the performance of DCNN classifier depends on data of the input joint groups.

The results showed that data from leg and trunk are the most important in the assessment of balance during walking. These results are expected because balance is related to body’s center of gravity, and leg and trunk play an important role in its position change. On the other side, human hands and upper limbs are usually moving freely during walking, thus when we exclude these data, the accuracy of the classification improved.

### 3.4 DCNN model interpretation with LRP

**FIGURE 7:**
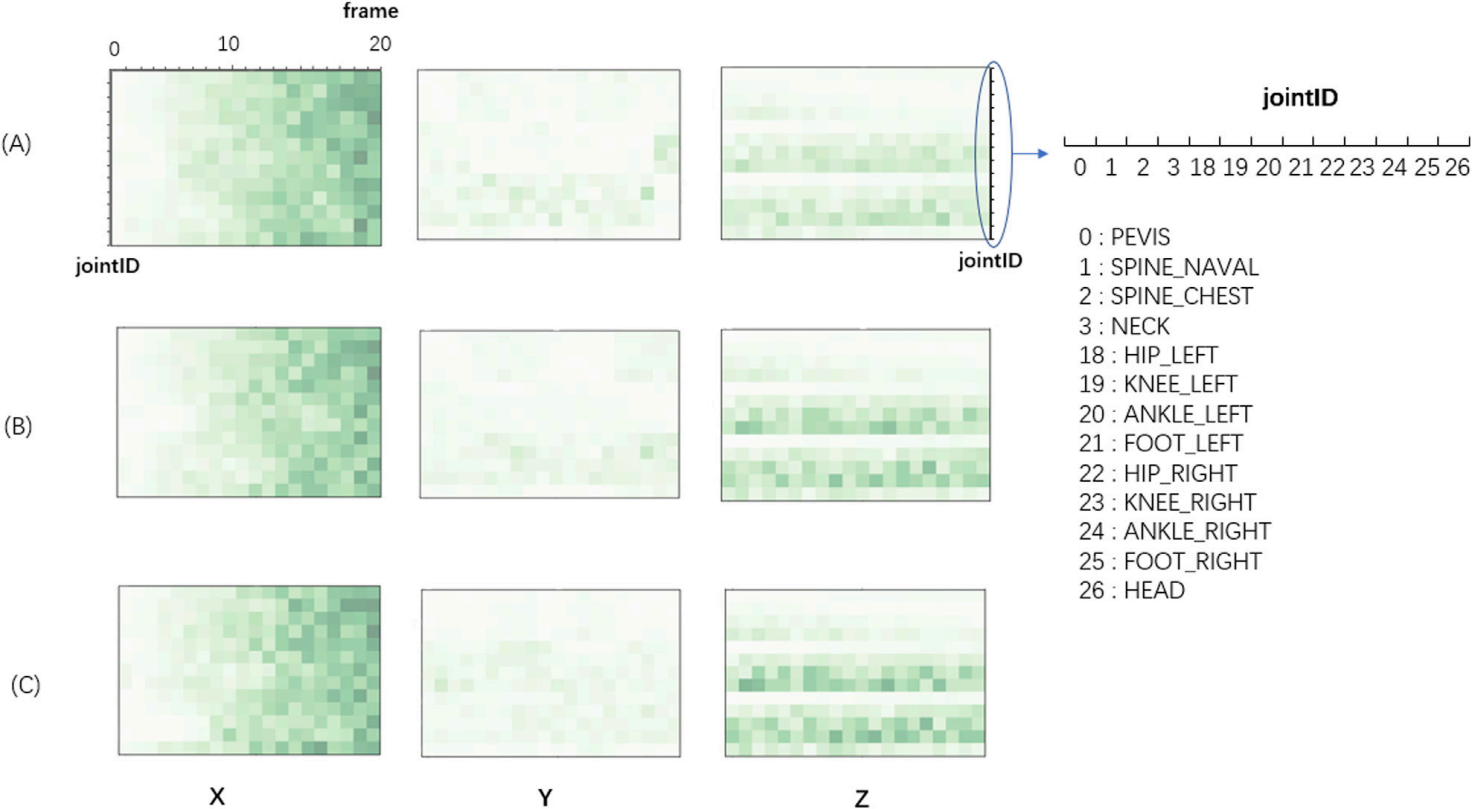
Color diagram of the contribution matrix of the sample input in the three levels of the subject. **(A)** Balance Level-0; **(B)** Balance Level-1; **(C)** Balance Level-2. The y-axis represents 13 joints in H4 skeleton node group and the x-axis represents 20 frames (time). The X refers to the front and back direction of the human body, the Y refers to the left and right direction, and the Z refers to the up-and-down direction.

We further used LRP technique to explain DCNN decisions in the balance assessment process as shown in Figure 7, which is the representation of data contribution matrix for H4 condition which has the best accuracy performance. The darker the color, the greater the value of the matrix.

In walking (X) direction, the matrix values gradually increased with time, which implies that the importance of data roughly increased with time. In reality, when a subject is walking, his/her position in X direction is gradually increase in X direction. The relationship of position and time is dependent on step size, walking speed and some other parameters that are widely used in popular balance assessment scales. The subjects with low balance ability have smaller walking speed, step frequency and step length, thus the distance difference between subjects with different balance levels gets bigger and bigger as time frames go on.

In left-right (Y) direction, the color of the entire Y block is much lighter than the color of X and Z blocks, suggesting left-right direction is the least important one for the classification task of balance levels. We believe that this is because the movement of the body in left-right (Y) direction during walking is relatively small, leading to a failure to recognize the left-right (Y) direction difference between different balance levels.

In the up-and-down direction (Z), lower limb data (left and right) have relative darker color, suggesting ankles and legs are the important parts for the classification task of balance levels. We believed that this is because the subjects with different balance abilities have different heights of foot lifting. In some assessment scales, lifting height of foot is also an important parameter to judge the level of human balance ability. The ankle joints and foot joints vary fast in the vertical direction during the lifting of foot. Additionally, the color of normal balance group (Level-0) was lighter than the other two, suggesting normal balance group is relatively stable and has less position changes than balance impaired groups (Level-1 and Level-2).

### 3.5 Comparison of the proposed DCNN with traditional machine learning methods

To validate the effectiveness of the proposed DCNN method, we compared the results of DCNN with those of traditional machine learning methods, such as Naive Bayes, Random Forest and support vector machines (SVM). We used an exhaustive grid search method to compute optimal values for hyperparameters of Naive Bayes, Decision Tree, and SVM to obtain the highest recognition accuracy for all three methods. Naive Bayes is used to fit the distribution of data samples, and gaussian naive Bayes is used here. For the three hyperparameters of random forest classifier, the number of weak classifiers, evaluation criteria and maximum depth, the grid search method is used to search for optimal values. The number of weak classifiers is {50, 100, 150, 200}, the evaluation criteria is {“entropy,” “gini”}, and the maximum depth is set to a range of 5–50. Random forest classifiers perform best when the number of weak classifiers is 50, the evaluation criterion is “entropy”, and the maximum depth is 24. SVM classifier has three important hyperparameter kernel types, kernel coefficients and penalty parameters. The kernel type is {“RBF,” “Linear,” “Poly”}, the kernel coefficient is {0.0001, 0.001, 0.01, 0.1, 1.0, 10.0}, and the penalty parameter is {1, 5, 10, 100, 1,000}. When the kernel type is Linear, the kernel coefficient is 1.0, and the penalty parameter is 1, the SVM classifier can achieve the best performance. In addition, we use two LSTM layers and one full connection layer to construct the LSTM network, where the number of neurons in LSTM layer is 32, and the number of neurons in full connection layer is 32.

Table 6 shows the comparison results of DCNN and different traditional machine learning methods. Among them, Naïve Bayes performed the worst, with accuracy of 73.33%, precision of 81.71% and F1 score of 73.37%. The accuracy of SVM is 86.66%, the precision is 87.96%, and the F1 score is 86.92%. The accuracy of Random Forest is 86.66%, the precision is 87.96%, and the F1 score is 86.81%. LSTM has 83.33% accuracy, 86.71% precision and 82.83% F1 score. Compared with these methods, the performance of DCNN classifier proposed by us is better. The improvement indicated the potential of the proposed DCNN in balance ability assessment.

**TABLE 6:** Performance of DCNN and different traditional machine learning methods.

Classifier	Accuracy	Precision	F1-score
DCNN	93.33	94.44	93.46
SVM	86.67	87.96	86.92
Random Forest	86.67	90.47	86.81
LSTM	83.33	86.71	82.83
Naive Bayes	73.33	81.71	73.37

Because DCNN can effectively extract low and high dimensional features, it has demonstrated better performance than traditional machine learning classifiers in many areas (Khagi et al., 2019; Mete and Ensari, 2019). DCNN and LSTM are often used to process image data and sequence data respectively. In this paper, we compared the two deep learning models in our experiments, and we found that the proposed DCNN model with residual structure has better performance than LSTM. The result is consistent with other previous literatures (Bai et al., 2018), in which word-level language are studied. With the help of residual architecture, one-dimensional convolutional networks can also make effective use of historical information. Therefore, in some cases, one-dimensional residual convolution networks can match or exceed the performance of LSTM.

### 3.6 Comparison of the proposed DCNN with commonly used CNN-based methods

To further validate the effectiveness of the proposed DCNN method, we also compared the results of DCNN with those of several commonly used CNN-based methods. These commonly used CNN-based methods including the AlexNet, the VGGNet and the ResNet-18. The hyperparameters of these three CNN-based networks were optimized to achieve better prediction accuracy.

Table 7 shows the comparison results of DCNN and different CNN-based methods. Among them, ResNet-18 performed the worst, with accuracy of 83.33%, precision of 88.89% and F1 score of 82.22%. The accuracy of AlexNet is 90.00%, the precision is 90.74%, and the F1 score is 89.95%. The accuracy of VGGNet is 86.67%, the precision is 90.48%, and the F1 score is 86.11%. Compared with these methods, the performance of DCNN classifier proposed by us is better. The architecture of the proposed DCNN is more suitable for the classification of walking balance ability task.

**TABLE 7:** Performance of DCNN and different CNN-based methods.

Classifier	Accuracy	Precision	F1-score
DCNN	93.33	94.44	93.46
AlexNet	90.00	90.74	89.95
VGGNet	86.67	90.48	86.11
ResNet-18	83.33	88.89	82.22

### 3.7 Comparing with posture description method

There are many studies that use Kinect for posture and motion recognition, but few studies that use Kinect to assess balance ability during walking. Although the goals of the two tasks are different, they are achieved using classification methods, so it can be considered to migrate the postures classification model to the walking balance ability classification.

To further validate the performance of the proposed method, we migrated and applied a state of art posture classification method proposed by Klishkovakaia et al. (Klishkovskaia et al., 2020). Briefly, the authors developed a low-cost and high-precision posture classification method based on posture description composed of vector lengths and angles. Movement can be described as a collection of postures. We can analyze differences in walking balance ability by establishing balance ability posture collections.

The performances of the reproduced method and the proposed DCNN method are shown in Table 8. We can see that the proposed model is better in the walking balance classification task. In the classification of postures task, the difference between postures is more obvious, such as walking and standing. However, in the classification of walking balance ability task, the postures are all walking postures. Therefore, the classification of walking balance ability may need more powerful feature extraction capability, which is the strong point of DCNN method.

**TABLE 8:** Performance of DCNN and posture description method.

Method	Accuracy	Precision	F1-score
Posture description method	86.67	87.68	86.43
DCNN	93.33	94.44	93.46

## 4 Conclusion

In this study, we used the Kinect 3D skeleton data and DCNN to assess balance abilities of human body during walking. Thorough parametric study including hyperparameters setting of DCNN and skeleton node selection was performed in order to obtain better performance. The proposed DCNN was compared with traditional machine learning methods, and the results showed that DCNN has the best performance. Our results suggest that 3D skeleton data and DCNN can be used for balance assessment with decent accuracy. The proposed method should be useful in early screening balance impaired people. It can partially replace commonly used balance measures and reduce the influence of subjective factors. In future work, we plan to further validate the proposed deep convolutional neural network model on 3D skeleton datasets from real patients. In addition, we plan to further subdivide the levels of walking balance ability to assess the patient’s situation more accurately. Moreover, we plan to use multiple Kinects in combination to increase data accuracy.

## Data Availability

The original contributions presented in the study are included in the article/Supplementary material, further inquiries can be directed to the corresponding author.
